# Ultrasound-Guided Percutaneous Balloon Aortic Valvuloplasty for Aortic Stenosis

**DOI:** 10.1155/2020/8086796

**Published:** 2020-03-16

**Authors:** Yedan Li, Kunjing Pang, Yao Liu, Muzi Li, Hao Wang

**Affiliations:** ^1^Department of Echocardiography, State Key Laboratory of Cardiovascular Disease, Fuwai Hospital, National Center for Cardiovascular Diseases, Chinese Academy of Medical Sciences and Peking Union Medical College, Beijing 100037, China; ^2^Department of Cardiovascular Surgery, State Key Laboratory of Cardiovascular Disease, Fuwai Hospital, National Center for Cardiovascular Diseases, Chinese Academy of Medical Sciences and Peking Union Medical College, Beijing 100037, China

## Abstract

Percutaneous balloon aortic valvuloplasty (PBAV), which is used to treat symptomatic aortic stenosis, requires ionizing radiation and contrast agent for imaging guidance. The aim of the study is to evaluate the feasibility and effectiveness of ultrasound-guided PBAV in patients with aortic stenosis. This case series included 30 patients (14 males; mean age, 61.5 ± 4.5 years) with moderate/severe aortic stenosis treated with ultrasound-guided PBAV at the Ultrasound Department, Fuwai Hospital, Beijing, China, between January 2016 and July 2019. Cardiac function (New York Heart Association grade) was assessed before PBAV and 1 month after the procedure. Aortic peak jet velocity, aortic valve orifice area (AVA), mean transvalvular pressure gradient (MTPG), left ventricular end-diastolic diameter (LVDD), left ventricular ejection fraction (LVEF), and left ventricular end-systolic diameter (LVESD) were determined before and immediately after PBAV using Doppler echocardiography. Preprocedural cardiac function was grade I in 3 cases, grade II in 9 cases, grade III in 10 cases, and grade IV in 8 cases. Postprocedural cardiac function was grade I in 22 cases, grade II in 4 cases, and grade III in 4 cases, suggesting that cardiac function was improved by PBAV. Ultrasound-guided PBAV resulted in significant improvements (*P* < 0.05) in aortic peak jet velocity (3.68 ± 0.811 m/s vs. 4.79 ± 0.63 m/s), MTPG (33.77 ± 13.85 mmHg vs. 54.54 ± 13.81 mmHg), AVA (1.96 ± 0.25 cm^2^ vs. 0.98 ± 0.12 cm^2^), LVEDD (51.90 ± 3.21 mm vs. 65.60 ± 6.81 mm), LVEF (63.46 ± 11.29% vs. 56.31 ± 11.04%), and LVESD (35.50 2.62 mm vs. 45.20 ± 2.42 mm). Ultrasound-guided PBAV is feasible and achieves good short-term effects in patients with aortic stenosis.

## 1. Introduction

Aortic stenosis is the most common valvular disease worldwide, and the vast majority of cases are acquired rather than congenital [1]. Rheumatic heart disease is an important cause of aortic stenosis in developing countries, whereas degenerative calcific aortic valve stenosis is the most common underlying cause in industrialized countries and tends to occur earlier in people with congenital bicuspid aortic valves or disorders of calcium metabolism [1–3]. The incidence of aortic stenosis increases with age from around 100 per 100,000 person-years in people aged 65–59 years to approximately 180 per 100,000 person-years in people aged ≥75 years [4], and the reported prevalence increases from 1.3% in those aged 60–69 years to 9.8% in those aged 80–89 years [5]. Although watchful waiting is advocated for asymptomatic patients, surgical intervention is required in symptomatic patients due to the increased risk of death [6]. The main therapies for aortic stenosis are surgical and include aortic dilatation and aortic valve replacement. Aortic valve replacement is generally suitable for patients who are tolerant of thoracotomy and extracorporeal circulation, and the two approaches available are surgical aortic valve replacement (SAVR) and transcatheter aortic valve replacement (TAVR) [7–9]. Patients with aortic stenosis who have poor cardiac function are often treated with aortic dilatation using techniques such as percutaneous balloon aortic valvuloplasty (PBAV). PBAV was first described by Lababidi in 1983 [10] and has since been used in the management of aortic stenosis. PBAV can be used to improve cardiac function in high surgical risk patients because it avoids extracorporeal circulation, and the technique has been applied successfully in the bridging of patients for definitive treatment with SAVR/TAVR or as a palliative therapy [11–19].

However, PBAV is not without complications and limitations [20]. One of the shortcomings of PBAV is the requirement for ionizing radiation-based imaging and contrast agents during the procedure, which can be particularly detrimental to pregnant women with congenital or acquired valvular disease and their fetuses [21–24]. The use of fluoroscopic techniques is also restricted in patients with allergy to iodine, aplastic anemia, and renal dysfunction. Thus, there is interest in the use of alternative approaches to guide PBAV that avoid the use of ionizing radiation or contrast agents. A few studies have reported the use of transesophageal or intracardiac echocardiography during PBAV [25, 26]. Furthermore, percutaneous ultrasound-guided balloon valvuloplasty in human fetuses has been described in a small number of cases [27, 28]. The outstanding advantage is that percutaneous ultrasound-guided balloon valvuloplasty was done without fluoroscopy.

We hypothesized that echocardiography could be used to guide PBAV. The aim of this case series was to evaluate whether ultrasound-guided PBAV might be a feasible and effective procedure in patients with aortic stenosis.

## 2. Materials and Methods

### 2.1. Ethical Approval

This case series was approved by the institutional review board of Fuwai Hospital (Beijing, China), which belongs to the Chinese Academy of Medical Sciences. Informed consent was waived because of the retrospective nature of the study.

### 2.2. Patients

This study enrolled 30 patients with aortic stenosis who underwent ultrasound-guided PBAV in the Ultrasound Department of Fuwai Hospital between January 2016 and July 2019.

The inclusion criteria were as follows [11]: (1) a diagnosis of moderate or severe aortic stenosis (aortic valve orifice area of <1.5 cm^2^) [29] was made based on the clinical manifestations and echocardiography; (2) color Doppler flow imaging showed that the aortic valve was still functioning; and (3) aortic valve replacement could not be performed due to severe hemodynamic instability or refusal by the patient, so the patient underwent PBAV; and (4) severe aortic stenosis with a peak Doppler gradient ≥70 mmHg or severe aortic stenosis with a peak Doppler gradient ≥50 mmHg accompanied by clinical symptoms and/or electrocardiogram (ECG) strain or heart failure.

Patients were excluded if any one of the following criteria was met: (1) active rheumatism or significant spinal deformity; (2) thrombosis in the left atrium; (3) supravalvular aortic stenosis or dysplastic aortic stenosis; (4) fibromuscular or tubular subvalvular aortic stenosis; and (5) PBAV was contraindicated in asymptomatic patients with aortic stenosis, a peak aortic gradient <40 mmHg and no ECG changes. Our hospital considered the procedure to be successful if the transvalvular pressure gradient of the dilated aorta decreased by ≥50% compared to that before the operation and the degree of reflux was below the median [30].

### 2.3. Ultrasound-Guided PBAV

**Figure 1:**
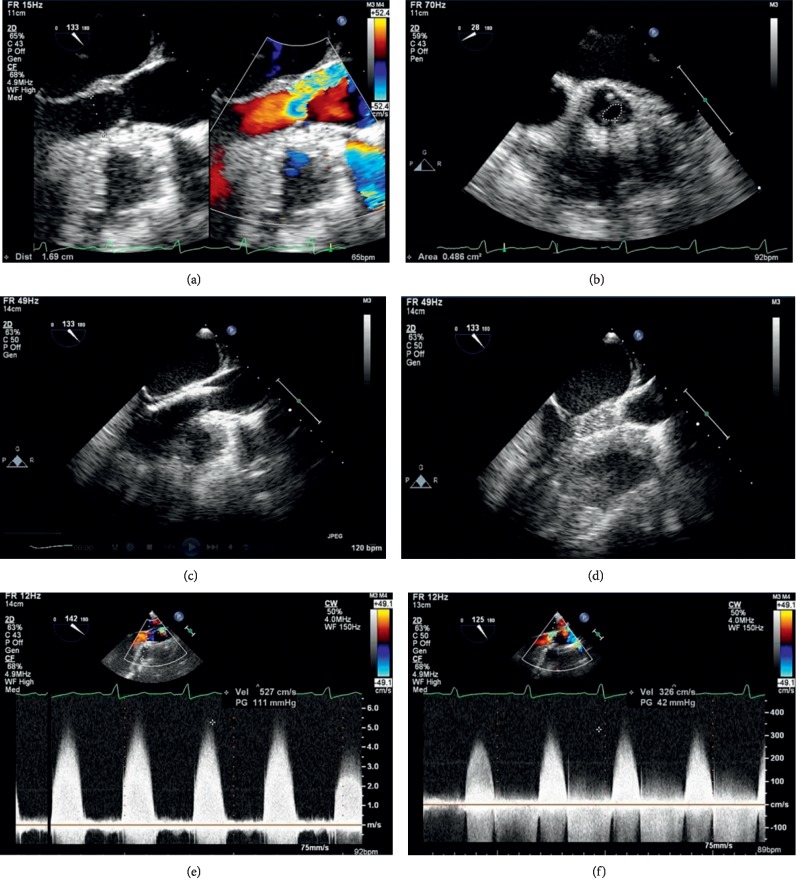
Representative images obtained from a patient during transthoracic ultrasound-guided percutaneous balloon aortic valvuloplasty. (a) Preoperative measurement of the aortic annulus (1.69 cm). (b) Preoperative measurement of the aortic valve area (0.48 cm^2^). (c) Intraoperative echocardiography showed that the guide-wire had passed through the stenotic aortic valve. (d) Intraoperative echocardiography showed that the balloon had passed through the stenotic aortic valve and the balloon had filled to expand the aortic valve. (e) The preoperative aortic valve flow rate was 5.3 m/s, and severe stenosis was evident. (f) Postoperatively, the aortic valve flow rate had decreased to 3.2 m/s and the aortic stenosis was significantly relieved.

A preliminary understanding of the type and severity of aortic stenosis was obtained from routine preoperative assessments, including physical examination, ECG, chest X-ray, and echocardiography. In general, the patients underwent CT aortic angiography before the operation to reduce the risk of abdominal aortic injury, because ultrasound cannot show that segment of the abdominal aorta clearly. The PBAV procedure was guided solely by ultrasound in all enrolled patients and conducted by a team of cardiologists, interventional physicians, and sonographers who all had more than 5 years of professional experience. Each patient was placed in the supine position, and local anesthesia was administered to the puncture site, which was then sterilized. After placement of a surgical drape, the region above the umbilicus was exposed to facilitate the ultrasonography examination. Echocardiography was performed using a Vivid i ultrasound system and 3S RS probe (GE Healthcare, Chicago, IL, USA) to generate the long-axis view of the left ventricle beside the sternum and an apical five-chamber view, and the most important is the suprasternal view. The right femoral artery was punctured, and a 10 F arterial sheath was placed. The guide-wire (Lunderquist Extra-Stiff Wire Guide, Cook, Inc) can only be shown under the guidance of ultrasound. The guide-wire was inserted, and a 6 F MPA2 catheter (Cordis) was guided by the guide-wire via aortic arch and ascending aorta to superior plane to aortic valve in suprasternal view, under the guidance of ultrasound, the catheter and guide-wire were advanced through the aortic orifice into the left ventricle, the 6 F MPA2 was then removed. Subsequently, a puncture needle was inserted through the implanted puncture cannula for puncture and injection of heparin sodium. The balloon (BALT, Montmorency, France) was advanced by using the guide-wire to the aortic valve, and the balloon diameter should not exceed that of aortic valve ring. The balloon dilator was expanded with normal saline. First, the front part of the balloon dilator was filled and the balloon is moved until it had reached the stenotic aortic valve. Then, saline was injected once again to inflate the balloon for dilatation of the valve. The saline in the balloon was extracted once echocardiography demonstrated that the aorta had been completely dilated by the balloon. Subsequently, the mobility of the aortic valve leaflets was monitored by ultrasound (apical five-chamber view). Dilatation was performed a further one or two times if the transvalvular pressure gradient across the aortic valve after dilatation had decreased by <50% of the value before the procedure. Representative images obtained during the procedure are shown in Figure 1.

### 2.4. Postoperative Management

The puncture site was compressed routinely to stop the bleeding after the operation. Blood pressure, heart rate, and the ECG were closely monitored. Postoperative ultrasonography was performed within 2 hours after the procedure. The arterial pulses in the lower extremity on the puncture side were observed closely to detect the occurrence of any possible complications.

### 2.5. Evaluated Parameters

Aortic jet velocity, aortic valve orifice area (AVA), mean transvalvular pressure gradient (MTPG), left ventricular end-diastolic diameter (LVEDD), left ventricular ejection fraction (LVEF), and left ventricular end-systolic diameter (LVESD) were measured before and immediately after the operation using Doppler echocardiography. Cardiac function was assessed using the New York Heart Association (NYHA) classification of cardiac functional capacity [31] before the operation and 1 month after the operation.

### 2.6. Statistical Analysis

The data were analyzed using SPSS 19.0 (IBM Corp., Armonk, NY, USA). Continuous data are presented as the mean ± standard deviation and compared between preoperative and postoperative time points using Student's paired *t*-test. Categorical data are expressed as frequency and percentage and were compared between groups using the chi-squared test. *P* < 0.05 was considered to be statistically significant.

## 3. Results

### 3.1. Baseline Characteristics of the Study Participants

A total of 30 patients (14 males and 16 females) with an average age of 61.5 ± 4.5 years (age range, 6–78 years) were enrolled in this study. The baseline clinical characteristics of the patients are shown in Table 1. Among the patients included in the analysis, 2 (6.6%) had allergy to iodine, 2 (6.6%) had renal dysfunction, 1 (3.3%) was pregnant, and 1 (3.3%) had aplastic anemia.

### 3.2. Comparison of Preprocedural and Postprocedural Cardiac Function

Cardiac function was evaluated before and after the operation using the NYHA classification. Preprocedural cardiac function was grade I in 3 cases, grade II in 9 cases, grade III in 10 cases, and grade IV in 8 cases, while postprocedural cardiac function was grade I in 22 cases, grade II in 4 cases, and grade III in 4 cases (Table 2). Pearson's chi-squared test indicated that NYHA grade was significantly lower after PBAV than before the procedure (*P* < 0.001), indicating that cardiac function was significantly improved by ultrasound-guided PBAV.

### 3.3. Comparison of Preprocedural and Postprocedural Echocardiographic Parameters

As shown in Table 3, ultrasound-guided PBAV resulted in significant improvements (all *P* < 0.05) in aortic peak jet velocity (3.68 ± 0.811 m/s vs. 4.79 ± 0.63 m/s), MTPG (33.77 ± 13.85 mmHg vs. 54.54 ± 13.81 mmHg), AVA (1.96 ± 0.25 cm^2^ vs. 0.98 ± 0.12 cm^2^), LVEDD (51.90 ± 3.21 mm vs. 65.60 ± 6.81 mm), LVEF (63.46 ± 11.29% vs. 56.31 ± 11.04%), and LVESD (35.50 ± 2.62 mm vs. 45.20 ± 2.42 mm).

### 3.4. Complications

There were no complications associated with the procedure such as vascular injury, cardiac tamponade, or systemic embolism.

## 4. Discussion

The main findings of the present case series were that cardiac function, aortic peak jet velocity, MTPG, AVA, LVEDD, LVEF, and LVESD were all significantly improved by ultrasound-guided PBAV. Our results indicate that ultrasound-guided PBAV is technically feasible and can achieve good short-term effects in patients with aortic stenosis. Notably, we successfully applied this nonfluoroscopic technique in patients who were pregnant or had allergy to iodine, aplastic anemia, or renal dysfunction. Thus, ultrasound-guided PBAV may be a good option for patients for whom conventional fluoroscopy poses a potential risk.

Numerous previous studies have concluded that PBAV, used either as a palliative therapy or as a bridge for definitive treatment with SAVR/TAVR, can improve cardiac function in patients with severe aortic stenosis. For example, it has been reported that PBAV can improve NYHA class [14, 15], MTPG [11–15, 17, 19], AVA [11–15, 17, 19], and LVEF [11, 14, 15, 17] both immediately after the procedure and at longer time points (e.g., 12 months). The results of the present study are thus entirely consistent with other published data using fluoroscopy-guided PBAV. Furthermore, prior clinical studies of patients with severe aortic stenosis have reported an adverse event rate of around 6–18% and a one-month mortality rate of around 3–17% [11–19], suggesting that PBAV has a sufficiently good safety profile for use in the treatment of patients with aortic stenosis who are not suitable for TAVR or SAVR.

Currently, PBAV is usually performed under X-ray guidance. However, X-ray imaging cannot clearly display the heart valves during surgery, which introduces a possible risk of excessive dilatation. Furthermore, X-ray imaging utilizes ionizing radiation and contrast agents that are potentially harmful to the patient, particularly if they are pregnant [23, 24]. In recent years, echocardiography has been increasingly used in place of X-ray-based imaging to guide percutaneous interventional techniques [32–34]. There have been a small number of case reports describing the use of percutaneous ultrasound-guided balloon valvuloplasty in human fetuses [27, 28], and a few studies have described PBAV-guided by transesophageal or intracardiac echocardiography [25, 26]. However, the use of only echocardiography to guide PBAV has not been reported previously. The present study demonstrated that ultrasound-guided PBAV is a feasible and effective technique because the procedure was completed successfully in all 30 patients. If the effectiveness and safety of our innovative approach are validated in additional larger-scale studies, the routine application of ultrasound during interventional techniques potentially could be expanded to include PBAV in patients with aortic stenosis.

The process of balloon dilatation is ideally observed from the long-axis views of the aortic arch, aorta, and left ventricle. The long-axis view of the aortic arch facilitates observation of catheter entry from the descending aorta into the ascending aorta and then the junction of the aortic sinuses. After the catheter enters the aortic sinuses, echocardiography should try to clearly display the stenotic aortic valve to facilitate the retrograde passage of the catheter through the valve into the left ventricle. The length of catheter displayed by ultrasonography is determined by the angle between the catheter and the sound beam, and longer catheter echoes can be displayed if the angle is small. The catheter is rarely perpendicular to the sound beam during the procedure, and thus a segment of the catheter can be displayed well in most cases. Usually, it is difficult to pass the balloon catheter through the aortic valve due to stenosis, contracture, and deformity of the valve. The length of balloon should cross the stenotic aortic valve without touching the mitral valve chordae, so the working guide-wire should be placed in front of the mitral valve device, and damage to the mitral valve chordae should be avoided. The positional relationship between the tip of the balloon and mitral valve device cannot be displayed using X-ray. By contrast, the position of the balloon can be adjusted more accurately under echocardiography, and the process of the balloon passing through the aortic valve and lodging at the valve orifice can be observed clearly. Moreover, perhaps the biggest advantage of echocardiography for guidance of PBAV is that the effects can be evaluated immediately after each dilatation without the use of ionizing radiation.

This study has some limitations. This was a case series at a single center and the sample size was small (only 30 cases), so it remains to be established whether the results are generalizable. The follow-up time was short, so longer-term outcomes were not assessed. No comparator group (for example, PBAV-guided by X-ray imaging) was included, so the relative advantages and disadvantages of transthoracic ultrasound guidance could not be evaluated. In addition, there was no formal safety analysis. Multicenter, larger-scale randomized controlled studies are needed to validate the effectiveness and safety of ultrasound-guided PBAV in patients with severe aortic stenosis.

## 5. Conclusions

In summary, the results of our case series indicate that PBAV guided by ultrasound alone is technically feasible and can achieve good short-term effects in patients with aortic stenosis.

## Figures and Tables

**Table 1:** Baseline characteristics.

Characteristics	Value
Male gender, *n* (%)	14 (46.7%)
Age (years), mean ± standard deviation (range)	61.5 ± 4.5 (6–78)
Weight (kg), mean ± standard deviation	42.30 ± 18.58
Bicuspid aortic valve, *n* (%)	10 (33.3%)
Hypertension, *n* (%)	10 (33.3%)
Current smoker, *n* (%)	8 (26.7%)
Diabetes mellitus, *n* (%)	5 (16.7%)
Previous PCI, *n* (%)	1 (3.3%)
Pregnancy, *n* (%)	1 (3.3%)
Allergy to iodine, *n* (%)	2 (6.6%)
Aplastic anemia, *n* (%)	1 (3.3%)
Renal dysfunction, *n* (%)	2 (6.6%)

PCI: percutaneous coronary intervention.

**Table 2:** Comparison of preprocedural and postprocedural cardiac function.

Variable	Before PBAV	After PBAV	*P*
NYHA cardiac function			<0.001
Grade I	3 (10.0%)	22 (73.3%)	
Grade II	9 (30.0%)	4 (13.3%)	
Grade III	10 (33.3%)	4 (13.3%)	
Grade IV	8 (26.7%)	0 (0.0%)	

Data are presented as *n* (%). NYHA: New York Heart Association; PBAV: percutaneous balloon aortic valvuloplasty.

**Table 3:** Comparison of preprocedural and postprocedural echocardiographic parameters.

Variable	Before PBAV	After PBAV	*P*
Aortic peak jet velocity (m/s)	4.79 ± 0.63	3.68 ± 0.811	<0.001
MTPG (mmHg)	54.54 ± 13.81	33.77 ± 13.85	<0.001
AVA (cm^2^)	0.98 ± 0.12	1.96 ± 0.25	0.031
LVEDD (mm)	65.60 ± 6.81	51.90 ± 3.21	0.038
LVESD (mm)	45.20 ± 2.42	35.50 ± 2.62	0.047
LVEF (%)	56.31 ± 11.04	63.46 ± 11.29	0.011

Data are presented as mean ± standard deviation. AVA: aortic valve area; LVEDD: left ventricular end-diastolic diameter; LVEF: left ventricular ejection fraction; LVESD: left ventricular end-systolic diameter; and MTPG: mean transaortic pressure gradient.

## Data Availability

The data used to support the findings of this study are available from the corresponding author upon request.
